# A note on the consistency of a behavioral play marker in piglets

**DOI:** 10.1186/2049-1891-4-33

**Published:** 2013-09-04

**Authors:** Wendy Mercedes Rauw

**Affiliations:** 1 Departamento de Mejora Genética Animal, Instituto Nacional de Investigación y Tecnología Agraria y Alimentaria, Crta de la Coruña km 7.5, Madrid 28040, Spain

**Keywords:** Behavior, Locomotor play, Pigs, Play marker, Welfare

## Abstract

**Background:**

Play behavior has been proposed as a new potential indicator of animal welfare. Animals play only if they are in a relaxed state. Play may improve adaptability to challenging environments which may be of interest in the breeding objective. Little information is available on play behavior in livestock species. The aim of the present study is to investigate whether play behavior in post-weaned piglets can be induced instantly in the presence of additional space and whether play markers are body weight, sex, and litter dependent. It is investigated whether playfulness is elicited by the moment or if this measure is consistent over different days.

**Methods:**

Thirty two piglets from four litters were released into a corridor and video recorded for eight min at 37, 41, 44 and 48 d of age. The first test was considered as an adjustment period and was not included in the analysis. In the second to the fourth test, joyful brusque movements (jumping, turning and running) were recorded with a camera and total number of movements (JOY#) and total time (JOYtime) were estimated individually. Animals were weighed at 41 d of age.

**Results:**

Females had higher scores of JOY# and JOYtime than males but this was significant only for JOY# at 41 d of age (*P* < 0.05). The effect of age was highly significant both for JOY# and JOYtime (*P* < 0.01). Animals with a higher JOY# and JOYtime also expressed a higher JOY# and JOYtime in the other tests, but this was significant only between 44 and 48 d of age (*P* < 0.01). Body weight at 41 d of age was not significantly related to JOY# and JOYtime in any of the tests.

**Conclusions:**

Locomotor play was induced instantly by the provision of increased space allowance. Litter origin had a significant effect on play behavior. In addition, locomotor play scores were consistent between two of the three tests.

## Background

Oliveira et al. [1] proposed assessment of play behavior as a new and promising potential indicator of animal welfare. There are evident emotions associated with play - joy and happiness - that drive animals into it [2]. Play in rodents is associated with and regulated by neurotransmitters that are known to play roles in other pleasurable activities (e.g., [3]). Indeed, animals play only if they are healthy, safe, well-fed and in a relaxed state, but not if they are under a stressful condition [4]. Therefore, play behavior can be used as an indicator of animal welfare.

Play behavior is categorized as social, object or locomotor play. Social play involves two or more animals and allows animals to develop social skills and to facilitate their integration into groups. Object play involves activity devoted to an inanimate object and is related to the development of motor skills. Locomotor play involves jumping, running and performing other motor activities in a sudden, persistent and frenetic manner. It helps with training and physical development by promoting anatomical and physiological benefits such as development and strengthening of bones and muscles and by increasing cardiopulmonary capability [1]. As given by Jensen and Kyhn [5], locomotor play, which includes vigorous jumping, kicking, and running, often interrupted by fast stops and turns in a new direction, can be performed by several animals at the same time, but does not involve physical contact. In contrast with social play or play fighting which looks very similar in appearance to real fighting, locomotor play does not have to be separated from a “serious”, non-playful, counterpart [6].

To date, very little information is available on play behavior in animal production systems. As indicated by Newberry et al. [7], piglets reared under intensive housing conditions may be unable to express their full repertoire of playful behavior due to limitations of space. The aim of this short investigation is to investigate whether play behavior in post-weaned piglets raised under intensive housing conditions can be induced instantly in the presence of additional space and whether play markers are body weight, sex, and litter dependent. In addition, it is investigated whether playfulness is elicited by the moment or if this measure is consistant over different days. For this purpose, piglets were released into a corridor and sudden, frenetic movements were investigated individually.

## Materials and methods

### Management

The pigs used in this experiment are part of a conservation program for Iberian pigs (of the breed Guadyerbas), which are kept in a semi-extensive management system. Females are kept outdoors where they are group mated. They are recovered before parity and kept indoors in individual farrowing pens, measuring 1.75 m × 1.75 m, from 5 to 6 d before parity until the litter is weaned. Heat lamps are used for the neonatal piglets. Sows are kept in farrowing crates to prevent them from crushing their piglets. Piglets are weighed at 21 and at 28 d of age and are individually earmarked at this time. At weaning, the sows are removed from the farrowing pen, but the litter remains in the farrowing pen for approximately 2 wk for minimizing the stress of weaning, change of diet and mixing. In addition, liquid food is provided in the pen at about 2 wk of age so that animals can (voluntarily) get familiar with this type of food before weaning. Since all males are used for reproduction (either at the farm or sold as such), male piglets are not castrated. Animals are inspected and cared for daily by the staff of the CIA Dehesón del Encinar where strict health protocols are followed. Sick and harmed animals are cared for by the veterinarian. The herd is a closed system with a maximum health status.

### Experimental set up

**Figure 1:**
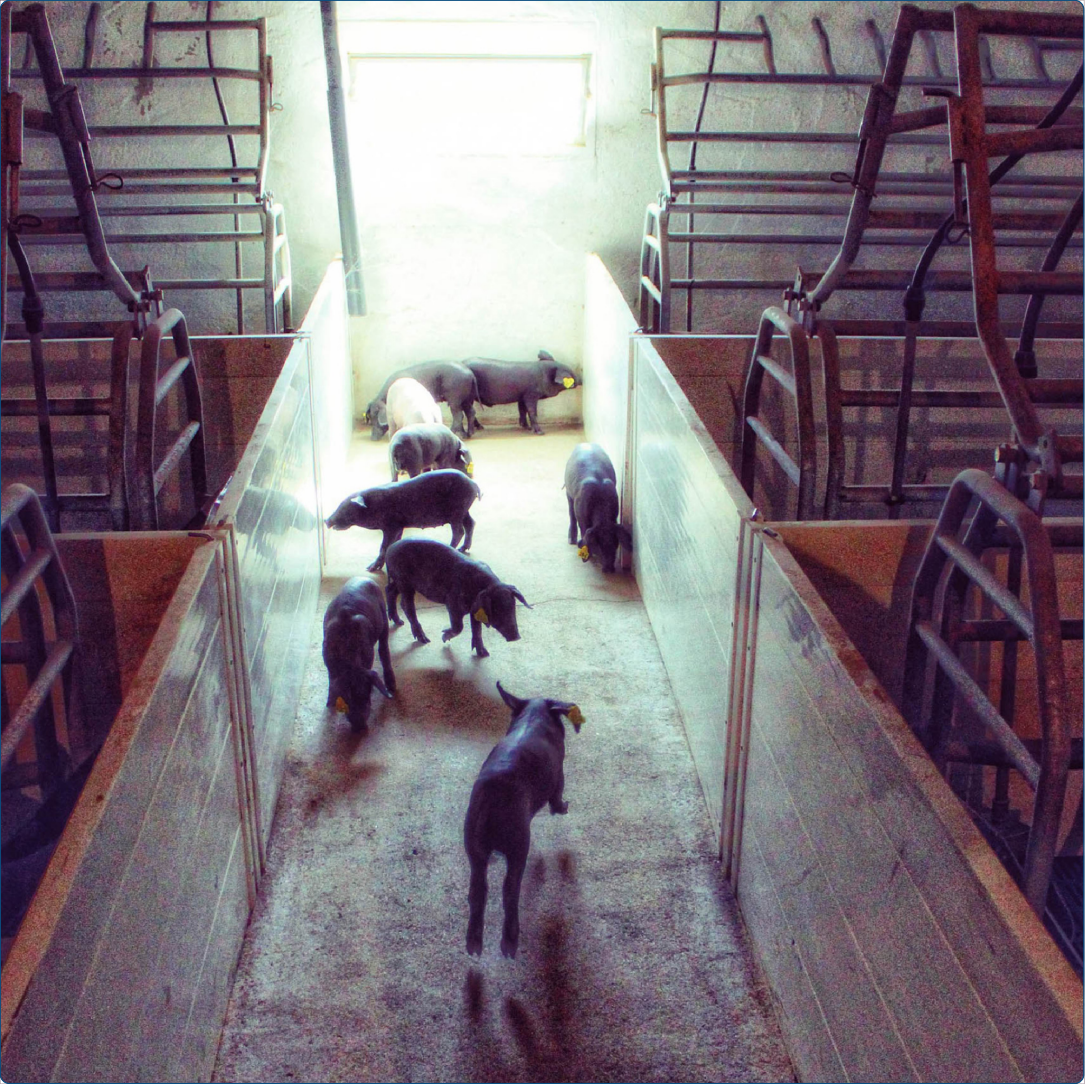
Experimental setup and example of a piglet displaying locomotor play.

In the present study, consistency of play behavior was investigated in 32 piglets (17 males and 15 females) from four litters (with 7, 8, 8, and 9 piglets, respectively) at 37, 41, 44, and 48 d of age; animals had been weaned at 35 d of age. Body weight (BW) was recorded at 41 d of age. Piglets were released into a corridor measuring 1.1 m × 5.3 m together with their litter mates for 15 min. Since play behavior appeared to be particularly exhibited in the first half of this period, play behavior was analyzed during the first eight min after release into the corridor. Since the animals had never been out of their pen before, the first test was considered as an adjustment period and was not included in the analysis. In the second to the fourth test, joyful brusque movements (jumping, turning and running) were recorded with a camera and the total number of movements and total time expressing these movements were estimated individually (JOY# and JOYtime, respectively). The experimental setup with a piglet displaying joyful movements is depicted in Figure 1. The experiments were approved by the INIA Scientific Ethic Committee Report CEEA2012/017, in agreement with the Spanish policy for animal protection RD1201/05 which meets the European Union Directives about the protection of animals used in experimentation.

### Data analysis

The SAS program was used for the statistical analysis of all traits [8]. The model used to analyse body weight at 41 d of age was:

(1) $$Y_{\mathrm{ijk}}=\mu +\mathrm{Litte}r_{i}+\mathrm{Se}x_{j}+e_{\mathrm{ijk}},$$

where μ = overall mean, Litter_i_ = effect of litter i (1 to 4; fixed class effect), Sex_j_ = effect of sex j (male, female; fixed class effect), and e_ijk_ = error term of animal k from litter i with sex j, e_ijk_ ∼ NID(0, σ^2^_e_). Body weight at 41 d of age tested by this model is denoted by Y_ijk_.

The traits at 41, 44 and at 48 d of age were analysed with a model for the analysis of repeated measures data with the procedure PROC MIXED. After analysis of several models within this procedure, data was analysed with the ‘autoregressive with heterogeneous variances model’ (ARH(1)) because it provided the best fit:

(2) $$Y_{\mathrm{ijkl}}=\mu +\mathrm{Litte}r_{i}+\mathrm{Se}x_{j}+\mathrm{Ag}e_{k}+e_{\mathrm{ijkl}},$$

where μ = overall mean, Litter_i_ = effect of litter i (1 to 4; fixed class effect), Sex_j_ = effect of sex j (male, female; fixed class effect), Age_k_ = effect of age k (41, 44 and 48 d of age; fixed class effect), and e_ijkl_ = error term of animal l from litter i with sex j at age k, e_ijkl_ ∼ NID(0, σ^2^_e_). Covariation within individuals was included in the RANDOM statement as the effect of animal l nested within litter i. All traits tested by this model are denoted by Y_ijkl_: JOY# and JOYtime.

Phenotypic correlations between JOY# and JOYtime at 41, 44 and 48 d of age were adjusted for the effects of litter and sex according to model (1). Since the effect of litter was not significant for the trait BW, correlations of BW where adjusted for the effect of sex only.

## Results

Table 1 presents least square mean values and standard errors of body weight at 41 d of age, and of the total number and total duration of joyful brusque movements in eight min at 41, 44 and 48 d of age, by sex and by litter. Male piglets tended to be heavier than female piglets but this difference was not yet significant (*P* = 0.0859). Total number of joyful movements was higher in female piglets than in male piglets but this was significant only at 41 d of age (*P* < 0.05). Total time expressing joyful movements was higher in female piglets than in male piglets but this was a tendency only at 41 d of age (*P* = 0.0692). The overall effect of litter was significant for the total number of joyful movements and for the total time expressing these movements (*P* < 0.01).

**Table 1:** Least square means and standard errors (SE) of body weight (BW) at 41 d of age (kg), and the total number (JOY#) and total duration (JOYt) of joyful brusque movements in eight min in three consecutive runway tests at 41, 44 and 48 d of age, by sex and by litter

**Items**	**Males**	**Females**	**Litter 1**	**Litter 2**	**Litter 3**	**Litter 4**
BW, kg	7.38 (0.23)	6.79 (0.25)†				
JOY#41 (#)	7.7 (1.3)	12.0 (1.4)*	11.9^**a**^ (1.6)	5.9^**b**^ (1.6)	11.5^**a**^ (1.6)	10.5^**ab**^ (1.8)
JOY#44 (#)	5.6 (1.4)	8.9 (1.5)	7.9^**a**^ (1.9)	8.5^**a**^ (1.9)	7.0^**a**^ (1.8)	6.1^**a**^ (2.1)
JOY#48 (#)	7.7 (0.9)	9.7 (1.0)	11.0^**a**^ (1.2)	10.0^**a**^ (1.2)	11.2^**a**^ (1.2)	2.2^**b**^ (1.3)
JOYt41 (s)	18.7 (5.1)	32.6 (5.5)†	54.0^**a**^ (5.7)	8.7^**b**^ (5.7)	24.3^**c**^ (5.3)	14.9^**bc**^ (6.0)
JOYt44 (s)	8.4 (2.5)	14.1 (2.6)	16.5^**a**^ (3.4)	11.7^**a**^ (3.4)	9.8^**a**^ (3.2)	8.3^**a**^ (3.6)
JOYt48 (s)	13.0 (1.9)	17.2 (2.0)	20.1^**a**^ (2.7)	18.7^**a**^ (2.7)	18.3^**a**^ (2.6)	2.9^**b**^ (2.9)

Effect of sex: * *P* < 0.05; † *P* < 0.10.

Effect of litter: a,b,c Values with different superscripts are significantly different (*P* < 0.05).

The effect of age was highly significant for the total number of joyful movements and the time expressing these movements (*P* < 0.01). Overall, piglets had a total of 9.8 ± 0.9, 7.2 ± 1.0 and 8.7 ± 0.7 joyful movements and expressed 25.4 ± 3.8, 11.3 ± 1.8, and 15.1 ± 1.4 s of joyful movements at 41, 44 and 48 d of age, respectively. JOY# was higher at 41 d of age than at 44 and 48 d of age (*P* < 0.05). JOYtime at 41 d of age was longer than that at 44 and at 48 d of age (*P* < 0.01) but JOYtime at 44 d of age was shorter than that at 48 d of age (*P* < 0.05).

Table 2 presents the correlation of the total number of joyful movements and the total time expressing joyful movements at 41, 44 and 48 d of age. Animals with a higher number and longer duration of joyful movements in the tests also expressed a higher number and longer duration of joyful movements in the other tests, but this was significant only between 44 and 48 d of age.

**Table 2:** Phenotypic correlations between the total number of joyful movements at 41, 44 and 48 d of age (above the diagonal), the total time expressing joyful movements at 41, 44 and 48 d of age (below the diagonal), and body weight at 41 d of age (BW)

**d of age**	**41**	**44**	**48**	**BW**
41	.	0.27	0.03	0.18
44	0.11	.	0.47**	0.06
48	0.19	0.43*	.	−0.14
BW	0.10	0.05	−0.22	.

* *P* < 0.05.

** *P* < 0.01.

**Figure 2:**
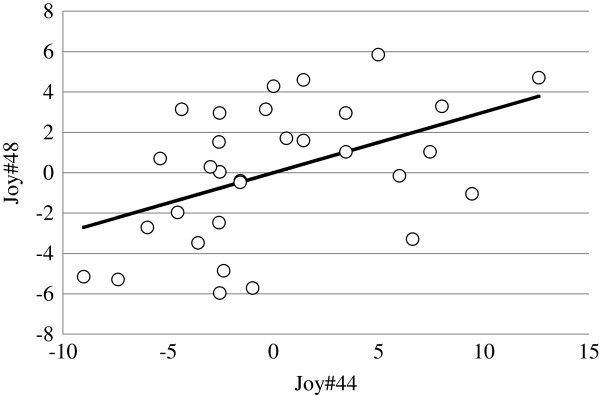
Phenotypic correlation between total number of joyful movements (JOY#), adjusted for the effects of sex and litter, at 44 and at 48 d of age.

The correlation of total number of joyful movements at 44 and 48 d of the age, adjusted for the effects of litter and sex, is presented in Figure 2. Body weight at 41 d of age was not significantly related to total number and total time expressing joyful movements in any of the tests.

## Discussion

Play behavior is related to the development of strength, motor coordination and physical resistance, learning of novel environmental information and creativity, and the acquisition of social skills. This enhances adaptive abilities so that they are prepared to handle future real-life situations [1]. Juveniles play to explore their environment and experiment with a variety of strategies that may be effective in that niche [9]. The main period of animal play coincides with the main period of physical, hormonal and social development, suggesting that play behavior can modulate developmental aspects [10]. In a review, Fromberg [11] describes children’s play as symbolic, meaningful, active, pleasurable, voluntary and intrinsically motivated, rule-governed and episodic. Allen and Bekoff [2] argue that social play, during which players follow the implicitly agreed upon rules not to dominate, prey on, or mate with their playmates, may provide a foundation of fairness for other forms of social cooperation and discuss turn-taking during social play as a behavioral indicator of rudimentary morality. According to these authors, cooperation and fairness can be driving forces in the evolution of sociality. Since play is a pleasurable emotion that only occurs when animals are in a relaxed (positive welfare) state it might be used as an indicator of animal welfare.

Enhancement of positive welfare and adaptive abilities may be possible by means of genetic selection. Whereas artificial selection has resulted in unprecedented increases in production traits, the possibility to include behavioral traits in selection programs is rarely considered, despite its potential to improve animal production and welfare. Breeding goals have been broadened beyond production traits in most farm animal species to include health and functional traits, but opportunities exist to improve breeding indices with the inclusion of behavior [12]. In an extensive review summarizing the estimates of genetic parameters for behavioral traits in cattle, pigs, poultry and fish, Canario et al. [13] indicate that the genetics of behavioral traits is understood to some extent, but is seldom accounted for in breeding programs. The rare genes identified that are associated with behavior are related to motivational processes, which clearly demonstrates that behavioral traits are important welfare indicators [13]. Breeding for behavior presents a number of particular challenges, and breeding for welfare even more. It is difficult and time-consuming to directly measure behavior in a consistent and reliable manner, necessary to evaluate the large numbers of animals that are necessary for a breeding program [12]. In addition, the major obstacle to overcome before genetic solutions can be implemented is determining which trait(s) to select for in order to truly improve animal wellbeing, either through direct measurements or through indirect measurements that are strongly correlated. This is particularly difficult as scientists have proposed many different conceptions and definitions of animal welfare, such as animal function, the balance of pleasure and suffering, and preference satisfaction [14,15]. Considering play behavior as a potential indicator of animal welfare has been proposed only recently [1].

When play markers are considered for inclusion in the breeding goal, it is important to investigate the reliability and consistency of several measures of play behavior in different contexts. In the present study, the increase in the space allowance increased the occurrence of locomotor play in piglets, which is in accordance with results by Jensen and Kyhn [5] in calves. The present experimental setup investigating locomotor play resulted instantly in an obviously pleasurable and measurable response and consistency of this response was measured at different ages.

Females had higher scores of total number of joyful movements and total time expressing joyful movements than males but this, respectively, was significant and approached significance only at 41 d of age. Sex differences in social play behavior in piglets were investigated by Dobao et al. [16], who observed a higher social play activity in males than in females. Social play included encounters between two individuals that are involved in reciprocal contact, such as pushing, butting, biting or mounting. Their results verify analogous results described in other species, which support the idea that contact-oriented play develops in part to provide the training for combatant skills in males [17]. Differences in play fighting between males and females have been shown to depend on the action of androgens perinatally [18]. However, as suggested by Berger [17], non-social locomotor play, as investigated in the present study, is likely part of a general anti-predator strategy in both sexes. Indeed, Sachs and Harris [19] observed that female lambs performed more locomotor play than male lambs and no sex differences were found in locomotor play in gazelle [20]. Newberry et al. [7] reported an absence of significant sex differences in the frequencies of the play markers “hop, scamper, pivot, toss head, shake object and carry object” in domestic piglets living in multi-litter groups with their dams.

In accordance with Jensen and Kyhn [5], in the present study, locomotor play generally did not involve physical contact and triggered locomotor play in a litter mate on few occasions only. However, the results of the present study show that the litter of origin had an effect on the measures of playful behavior. Piglets from one of the litters generally appeared to play for a particularly long time, while piglets of another litter generally appeared to play for a particularly short time. These results indicate that the litter environment or ‘a general mood’ in the litter may affect all litter mates during play; further research is needed since the present study included four litters only. In addition, it may indicate that locomotor play has a genetic component. Walker and Byers [21] indicated a possible genetic basis for locomotor play in house mice with a heritability of 0.55 ± 0.40. Heritability of play behavior in farm animals needs to be investigated when this trait is considered to be included in the breeding goal.

Litten et al. [22] observed a significant relationship between body weight and behavioral development in piglets. Piglets that are heavier at birth gain more weight and are the more successful fighters among piglets of a litter [23]. Sundman [24] observed that piglets suckling at the anterior teats tended to be more playful, and piglets suckling at the rear teats less playful, than those suckling the middle teats. In the present study, body weight adjusted for sex was not significantly related to the number or time of joyful movements in any of the three tests. This may indicate that the joyful movements investigated in this study are relatively unrelated to the piglet’s social position within the litter.

When play behavior is considered as a measurement that can be included in the breeding objective it is important that a play marker is chosen that can be measured instantly and reflects the inherent play behavior of the individual when other factors are kept constant. In the present study, the phenotypic correlations between total time at 41, 44 and 48 d of age, adjusted for the effect of litter and sex, were mostly positive, although significant only between 44 and 48 d of age; the same was true for the total number of movements. These results indicate that playfulness may be a trait that is inherent to the individual but still much dependent on the day of measurement. Newberry et al. [7] and Jensen and Kyhn [5] indicated that play behavior decreased with age. Indeed, on average in the present study, the total number and total time exhibiting locomotor play was highest at 41 d of age. This may be an effect of getting familiar with the test situation also.

In the present experiment, play behavior has been investigated in piglets that were weaned at 35 d of age. Weaning at this age is not common in commercial production animals where animals are commonly weaned around 21 d of age or earlier [25]. Under commercial conditions, piglets are regrouped and mixed with unfamiliar pigs resulting in aggressive and submissive agonistic behavior [26]. It will be of particular interest to investigate the relationship between pre-weaning play behavior and post-weaning adaptive abilities that are required during mixing. Donaldsen et al. [27] investigated the effects of early play experience on play behavior of piglets after weaning where they hypothesized that play experience gained by piglets during early ontogeny would affect the ability to cope with weaning stress. Indeed, piglets that had been allowed to play before weaning regained frequency of play behavior sooner after weaning [27]. Future research will investigate whether pre-weaning play behavior is related to learning ability and adaptability in different environments.

## Conclusions

Since play is a pleasurable emotion that only occurs when animals are in a positive welfare state it might be used as an indicator of animal welfare. In addition, since play behavior may improve flexibility and adaptability to challenging environments it may be of interest to include a measure of play in the breeding goal. Locomotor play investigated in this short investigation is an obviously pleasurable and measurable response that was induced instantly by the provision of increased space allowance. Litter origin had a significant effect on play behavior, in addition, locomotor play scores were consistent between two of the three tests. It remains to be investigated whether this trait is heritable in pigs and how it relates to learning ability and adaptability.

## Competing interests

The author declares that there is no competing interest.
